# Facile synthesis of N, P-doped carbon dots from maize starch *via* a solvothermal approach for the highly sensitive detection of Fe^3+^†

**DOI:** 10.1039/d0ra06209j

**Published:** 2020-09-10

**Authors:** Guohua Dong, Kun Lang, He Ouyang, Wenzhi Zhang, Liming Bai, Shijie Chen, Zhuanfang Zhang, Yueyue Gao, Zhonghua Mu, Xiaodan Zhao

**Affiliations:** College of Chemistry and Chemical Engineering, Heilongjiang Provincial Key Laboratory of Catalytic Synthesis for Fine Chemicals, Qiqihar University Qiqihar 160006 P. R. China wenzzhang1968@163.com; Key Laboratory of Photovoltaic Materials, Henan University Qiqihar 151001 P. R. China; College of Environmental and Chemical Engineering, Hefei University of Technology Hefei 230009 P. R. China

## Abstract

Nitrogen/phosphorus-doped carbon dots (N, P-CDs) with a quantum yield as high as 76.5% were synthesized by carbonizing maize starch *via* a facile ethanol solvothermal approach. Transmission electron microscopy (TEM) measurement shows that the as-prepared N, P-CDs displayed a quasi-spherical shape with a mean size of *ca.* 2.5 nm. Fourier transform infrared spectroscopy and X-ray photoelectron spectroscopy disclosed the presence of –OH, –NH_2_, –COOH, and –CO functional groups over the surface of N, P-CDs. On the basis of excellent fluorescent properties with strong blue fluorescence emission at 445 nm upon excitation at 340 nm, these N, P-CDs were adopted as a fluorescent probe towards the effective detection of Fe^3+^ ions in water. The limit of detection (LOD) was as low as 0.1 μmol L^−1^ and showed a better linear relationship in the range of 0.1 ∼ 50 μmol L^−1^. In conclusion, these synthesized N, P-CDs can be efficiently used as a promising candidate for the detection of Fe^3+^ ions in some practical samples.

## Abstract 2

Nitrogen/phosphorus-doped carbon dots (N, P-CDs) with a quantum yield as high as 76.5% were synthesized by carbonizing maize starch *via* a facile ethanol solvothermal approach and utilized for the detection of Fe^3+^.

## Graphical abstract

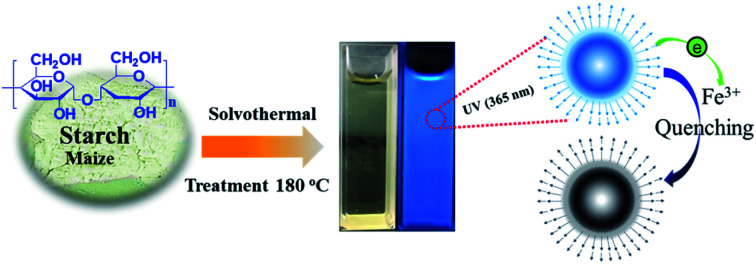

## Introduction

Carbon dots (CDs) are a category of promising nanomaterials with sizes below 10 nm. They have displayed tremendous research value for chemosensors, energy-storage electrocatalysis, photocatalysis and live cell imaging due to their intrinsic properties, such as tunable fluorescence emission, biocompatibility, low-cost synthesis and prominently low cytotoxicity. Therefore, CDs are regarded as a better replacement for heavy metal based quantum dots.^1–4^ Conventionally, there are two main strategies for the preparation of CDs.^5,6^ The first method, also named the top-down method, is conducted through breaking down carbon precursor materials with larger molecular structures, such as graphite, carbon nanotubes, and graphite oxide *via* laser ablation, electrochemical oxidation, ultrasonication, *etc.*^1,7^ The other method is called the bottom-up route, which is performed through combustion, hydrothermal, microwave pyrolysis, microwave and so on.^1,8^ Regardless of the techniques applied for the synthesis of CDs, the selection of the starting carbon source materials is one of the prerequisites.

For the green synthesis of CDs, one of the cost-effective and eco-friendly carbon sources is the natural biomass materials, such as various fruit juices, grass, plant leaves, chitosan and sweat pepper, kitchen wastes, *etc.*^3,8–12^ However, for most of the synthesis of CDs, this type of green materials commonly suffers from serious bottleneck such as time consuming process, toxic organic solvents, and especially the low fluorescence quantum yield of the final CDs, *etc.*^3,13,14^ Therefore, for improving the fluorescence properties, many efforts have been devoted to the modulation and optimization of the preparation routes of CDs derived from the natural biomass materials, such as doping with other functional materials and surface modification and passivation. Among them, heteroatoms doping is a facile and effective strategy for further modulating the fluorescence properties of the biomass-based CDs. For example, N doped CDs can be prepared by utilizing both of the folic acid and Chinese yam as the source of carbon and nitrogen, respectively.^15^ Meanwhile, the fluorescence quantum yield as high as 25% can be confirmed for the co-doped CDs with nitrogen and sulfur (abbreviated as N, S/C-dots).^16^

Similar with other metal ions such as Na^+^, K^+^, Ca^2+^, Zn^2+^ and Cu^2+^, Fe^3+^ ion is one of the most necessary elements in human body and other biological systems.^17,18^ Its excess or deficiency will result in different biological disorders, such as the eonian loss of motor skills and Parkinson's and Alzheimer's diseases, *etc.*^17,19^ Thus, it is extremely necessary for periodically monitoring the Fe^3+^ or other metal ions to investigate the physiological functions or diagnose and prevent diseases. In the past few years, many effective techniques have been reported for recognizing the ions with low concentrations, for instance gold nanoparticles, electrochemical methods, photonic crystals, luminescence, holography, *etc.*^17,20^ However, these techniques commonly suffer from many undesired disadvantages, such as high-cost of the raw materials, complexity of the material fabrication, inflexible use and detailed data analysis and so on. Actually, the above-mentioned CDs with many fascinating properties such as bio-compatibility, low-cytotoxity, good dispersibility in any solvents and photostability can be the perfect candidate for the selective and sensitive detection of the Fe^3+^.^21–23^

Maize is the third most important worldwide agricultural crop species for satisfying the human and animal nutrition requirements. Thus, there are enough raw materials for the exploration of the CDs together with its potential application. Herein, we developed a scalable and facile synthesis of nitrogen and phosphorus co-doped CDs (N, P-CDs) *via* the alcohol solvothermal method from widespread maize starch. TEM measurement demonstrated that the synthesized N, P-CDs showed quasi-spherical shape with mean size of *ca.* 2.5 nm. Fourier transform infrared spectroscopy and X-ray photoelectron spectroscopy revealed the presence of –OH, –NH_2_, –COOH, and –CO functional groups over the surface of N, P-CDs. Furthermore, the N, P-CDs displayed excellent fluorescent properties with strong blue fluorescence emission at 445 nm upon excitation at 340 nm. Finally, the N, P-CDs were adopted as a fluorescent probe towards the effective detection of Fe^3+^ ions in water by fluorescence spectroscopy. The limit of detection (LOD) was as low as 0.1 μmol L^−1^, which is far lower than that of the concentration value proposed by World Health Organization (WHO).

## Experimental

### Chemicals

Disodium hydrogen phosphate dodecahydrate (Na_2_HPO_4_·12H_2_O), sodium dihydrogen phosphate hydroxide (NaH_2_PO_4_·2H_2_O), corn starch, urea, CuSO_4_·5H_2_O, NiSO_4_·6H_2_O, Al_2_(SO_4_)_3_·18H_2_O, K_2_SO_4_, FeCl_3_·6H_2_O, CaCl_2_, BaCl_2_, Hg(NO_3_)_2_, Cd(NO_3_)_2_·4H_2_O and ZnSO_4_·7H_2_O were purchased from Tianjin Kaitong Chemistry Co., Ltd., China. MnCl_2_·4H_2_O were bought from Fuchen Chemical Reagent Co., Ltd. (Tianjin, China). Dialysis bags (molecular weight cut off = 3500) were purchased from the Chemical Reagent Co., Ltd., China. Note that all the chemicals used in this work were analytical grade without further post-treatment and the used water is Mili-Q ultra-pure water processed in our lab.

### Synthesis of N, P-CDs

**Fig. 1:**
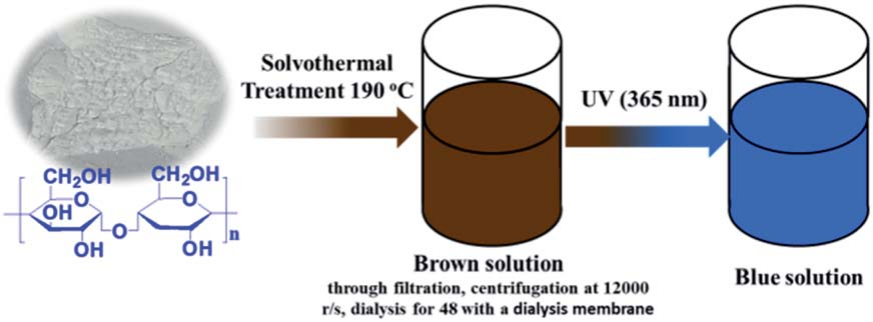
Schematic illustration of synthesis of N, P-CDs from maize starch *via* solvothermal treatment and its visible blue emission under UV of 365 nm.

Corn starch (1.5 g), Na_2_HPO_4_·12H_2_O (0.35 g), NaH_2_PO_4_·2H_2_O (0.35 g) and urea (0.7 g) were dispersed into absolute ethyl alcohol (18 mL) and energetically stirred for 10 min in a Teflon-lined stainless-steel autoclave. After heating at 190 °C for 12 hours and then cooling down to the room temperature, the resulting dark brown solution was collected with a centrifugal process at a speed of 10 000 rpm. The final supernatant was dialyzed through a dialysis membrane (molecular weight cutoff is 3500) against Mili-Q ultra-pure water for 2 days with changing the solvents every 12 h. At last, the brown N, P-CDs liquid was obtained. For characterization, the N, P-CDs powder was obtained *via* evaporating the obtained solution with a rotary evaporator. The detailed synthetic procedure was schematically presented in Fig. 1.

### Detection Fe^3+^ by N, P-CDs

The selectivity measurements for metal ions were as following: 1 mL different metal ions stock solution (Fe^3+^, Cu^2+^, Ba^2+^, Ca^2+^, Zn^2+^, Al^3+^, Cd^2+^, Mn^2+^, K^+^, Hg^2+^) in Mili-Q ultra-pure water were added into 9 mL of N, P-CDs solutions and stirred for 10 min at room temperature for obtaining the mixture solutions with a uniform metal ions concentration of 50 μmol L^−1^, respectively. Then the fluorescence emission spectra of the above-formed mixture solutions were recorded and compared. Additionally, aiming at the sensitivity characterization, a series of Fe^3+^ stock solutions (1000 μmol L^−1^) with different volume were initially added into 10 mL N, P-CDs solution and then supplemented some Mili-Q ultra-pure water into the above-mentioned solutions for keeping a uniform concentration of N, P-CDs. The fluorescence emission spectra of the resulted solutions were recorded after reaction for 10 min at room temperature. It was noted that the final Fe^3+^ concentrations of the mixture solutions are varied from 0 μmol L^−1^ to 500 μmol L^−1^ and the optimal diluted N, P-CDs aqueous was utilized as the assay of sensitivity and selectivity measurements.

### Characterizations

The morphology of N, P-CDs was characterized by transmission electron microscopy (TEM JEOL-2100 F) with accelerating voltage of 200 kV. The Raman spectra were obtained *via* a Raman spectrometer (Renishaw, Britian) with a synapse CCD detector, and the spectrograph uses 600 g mm^−1^ gratings and a 532 nm He–Ne laser. The UV-vis absorption spectra were recorded on a Puxi TU-1900 UV-vis spectrophotometer. Fluorescence spectra measurement were performed on a Shimadzu RF-530PC spectrophotometer. The Fourier transform infrared (FT-IR) spectra were collected on a Thermo Nicolet 380 instrument with the scanning range from 4000 cm^−1^ to 500 cm^−1^ and the KBr tablet was used for holding the dried CQDs powder sample. The X-ray photoelectron spectroscopy (XPS) was performed with Thermo ESCALAB250Xi by Al Kα monochromatic X-ray radiation. Fluorescence emission and life decay spectra were collected in a FLS 920 luminescence spectrometer (Edinburgh, German). A well-known comparative method was adopted to determine the QY of the N, P-CDs. Specifically, quinine sulfate (QY of 54%) was utilized as a reference and the QY for the N, P-CDs was calculated by the following expression:^3,14^
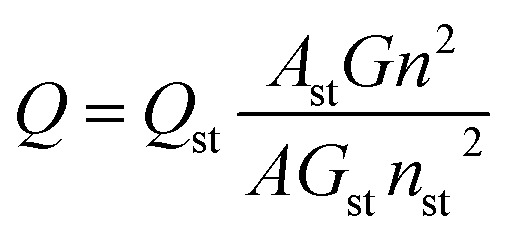
where *Q* denotes the QY, *A* is the absorbance intensity value recorded at the excitation wavelength of 340 nm, *G* is the measured integrated emission intensity in the wavelength range 380–700 nm and *n* represents the refractive index (1.33 for water). Because the quinine sulfate was dissolved in 0.1 M dilute H_2_SO_4_, the value of *n*/*n*_st_ is approximately equal to 1.0. The subscript st represents the standard quinine sulfate. Additionally, the optical absorbance intensity values of N, P-CDs and quinine sulfate should be in the range of 0–0.1 at 340 nm for avoid any significant reabsorption deviation.

## Results and discussion

**Fig. 2:**
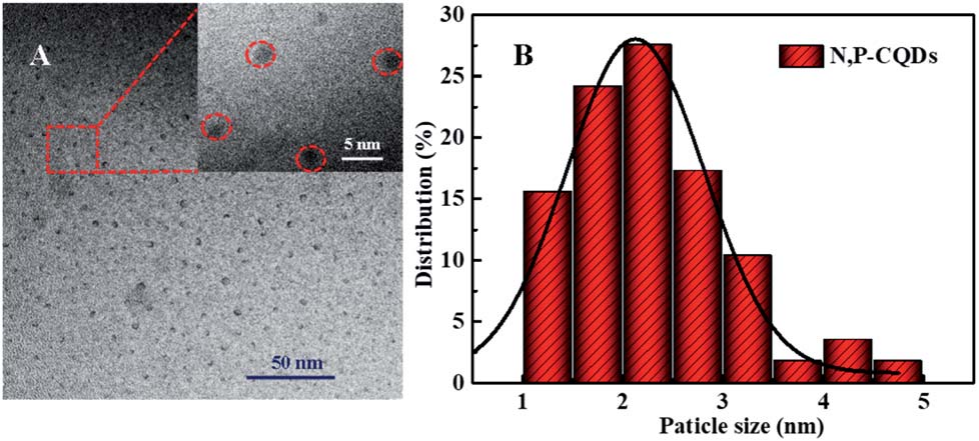
(A) The TEM image and (B) the statistical histogram of the particle size distribution for the synthesized N, P-CDs, inset of Fig. 1A is the magnification of TEM image.

Commonly, CDs originated from the bio-mass carbon source exhibit relatively poor fluorescence properties due to its lower heteroatom (such as N) content in bio-mass molecular structure.^24–26^ Thus, with the aiming at obtaining highly fluorescent CDs by introducing much more heteroatom, we synthesized N, P-CDs with a facile solvothermal route by utilizing maize as the bio-mass carbon source and urea together with phosphate as the N and P source, respectively. After purifying *via* a process of successive filtration, centrifugation and dialysis for the solvothermal treated production, the N, P-CDs with brightly blue photoluminescence emission under UV (365 nm) and well mono-dispersion was successfully obtained. After measurement and calculation, the formed N, P-CDs has a QY value as high as 76.5%, which is relatively higher than that of the literatures' reports. The morphology of the above-obtained N, P-CDs was investigated by TEM measurement and the result was displayed in Fig. 2. As shown in Fig. 2A, the N, P-CDs clearly displays a morphology of quasi-spherical shape, and these N, P-CQDs are well dispersed in the solvent, which is nearly in agreement with the other CQDs demonstrated in previous literature.^27–29^ The statistical histogram based on the TEM image analysis shown in Fig. 2B revealed that the N, P-CDs exhibits a narrow particle size distribution range from 1 nm to 5 nm with an average diameter of *ca.* 2.5 nm.

**Fig. 3:**
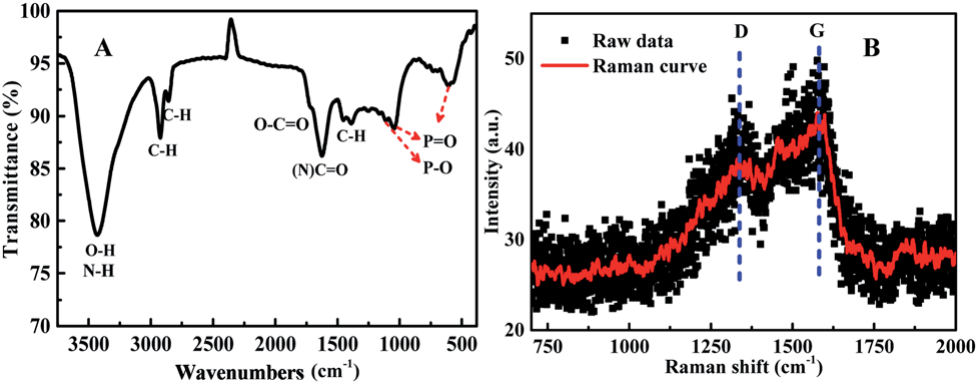
(A) The FT-IR and (B) Raman spectrum for the synthesized N, P-CDs.

The surface functional groups of the synthesized N, P-CDs were investigated by FT-IR spectroscopy. As illustrated in Fig. 3A, the band at 3025–3674 cm^−1^ is broad and strong, which might be ascribed to the overlap of –OH/–NH stretching bands. Otherwise, a sharp band at 2924 cm^−1^ accompanied with a shoulder band at 2850 cm^−1^ was observed, which should be assigned to the C–H stretching of CC moiety present in the core of the N, P-CDs. The vibrational absorption peaks at 1623 and 1719 cm^−1^ corresponded to the (O, N)–C=O/C=C stretching vibration, confirming the presence of an unsaturated aromatic rings shaped with graphitic structure. Moreover, the absorption peaks around 607 cm^−1^, 1047 cm^−1^, 1119 cm^−1^ are devoted to the phosphate group (P=O or P–O). The result confirmed the synthesized N, P-CDs were packaged with different functional groups, which could improve the aqueous solubility of N, P-CDs for potential applications in the bio-imaging and sensor fields.^30,31^ Moreover, as illustrated in Fig. 3B, two peaks with low intensity can be easily observed in the Raman spectrum of the prepared N, P-CDs. The peak at around 1580 cm^−1^ (G band) are attributed to the vibration of sp^2^-bonded ordered graphite carbon atom in a two-dimensional hexagonal lattice. Besides, the peak at about 1340 cm^−1^ (D band) is related to the vibrations of sp^3^-bonded (dangling bonds) carbon atoms in the peripheral plane of the disordered graphite or glassy carbon.^32–34^ Therefore, the Raman results suggest that the synthesized N, P-CDs have aromatic graphitic structure with minor surface defects.

**Fig. 4:**
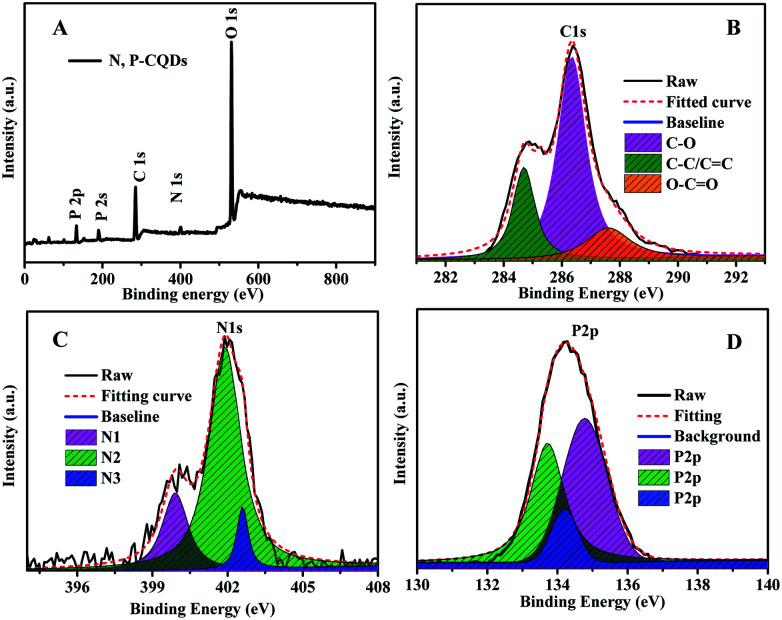
(A) The XPS survey spectrum (B) the C1s (C) N1s and (D) P2p XPS spectra of the synthesized N, P-CDs.

The functional groups and composed elements of the N, P-CDs were further revealed by the XPS measurements. As depicted in Fig. 4A, we can clearly observe the peaks of P2p, P2s, C1s, N1s and O1s at 133.1 eV, 189.4 eV, 284.6 eV, 400.3 eV and 531.0 eV, respectively, indicating that the N, P-CDs is primarily composed of four types of elements including C, P, O, N. Undoubtedly, the N and P mainly be resulted from the heteroatom doping with urea and phosphate reaction precursors. Additionally, from the high-resolution XPS spectra of N1s, P2p and C1s shown in Fig. 4B–D, we can see that each of them can be divided into multiple peaks, which are ascribed to multiple kinds of chemical bonds. For example, the C1s exists as the states of C–C/C=C, C–N/C–O, C=O–N, as well both of the XPS spectra of N1s and P2p consist of three peaks, behaving three types of the existence states in the N, P-CDs.^9,15,18^

**Fig. 5:**
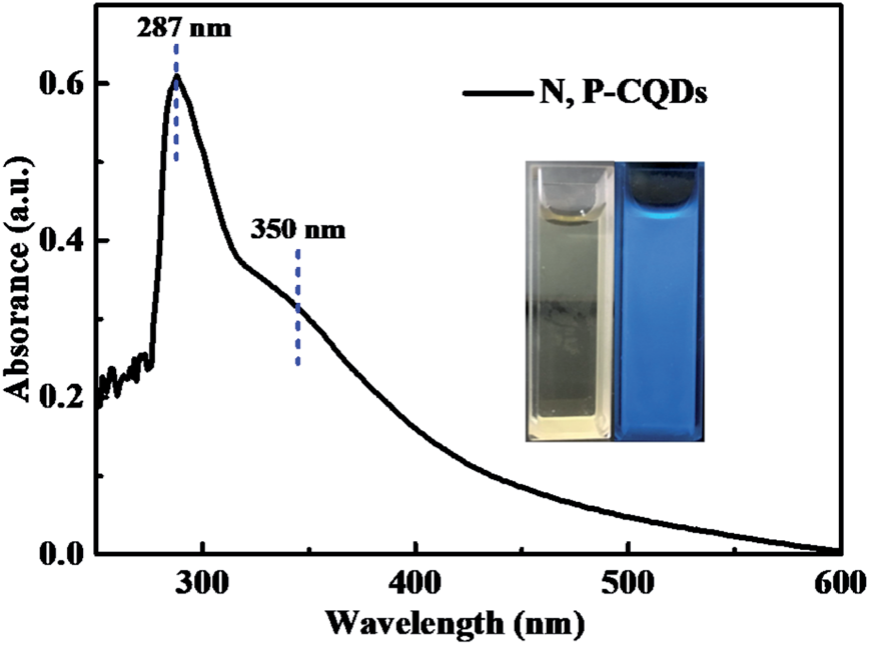
The absorption spectrum of the synthesized N, P-CDs, inset is the photographs of the N, P-CDs solution in ethanol irradiated under sunlight (yellow) and UV light (blue).

The optical property of the N, P-CDs was initially investigated by performing the UV-vis absorption spectroscopy measurement. As displayed in Fig. 5, the absorption spectrum curve of N, P-CDs displays two peaks centered at 287 nm and 350 nm, respectively. As demonstrated in many previous works, the obvious absorption peak located at 287 nm likely be assigned to the π–π transition of sp^2^ C=C in aromatic conjugate domains, which is the characteristic of synthesized CDs.^35,36^ Another peak at 350 nm may be associated with the n–π* transitions of C=O or others.^27^ Additionally, we can also observe from the inset of Fig. 5 that the N, P-CDs solution exhibits pale yellow color under sunlight and in contrary bright blue color irradiated with the UV light (365 nm), indicating that this N, P-CDs has significant photoluminescence property.

**Fig. 6:**
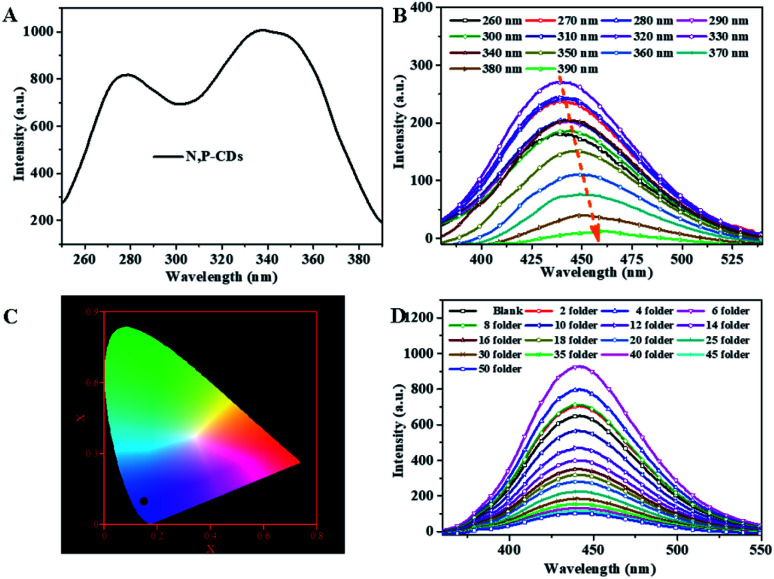
(A) The excitation spectra (B) the corresponding fluorescence emission spectra (C) CIE image of the N, P-CDs. (D) The emission spectra of N, P-CDs solution with different dilution multiple with H_2_O excited at 340 nm.

Sequentially, the fluorescence property of the N, P-CDs was studied by the photoluminescence (PL) spectroscopy. As shown in Fig. 6, no matter what excitation wavelength was selected range from 260 nm to 390 nm (Fig. 6A), the N, P-CDs wholly displays obvious strong PL emission in the region of around 360 nm to 550 nm (Fig. 6B). Furthermore, with the excitation wavelength increasing from 260 nm to 390 nm, the PL emission peak of the N, P-CDs shows a tiny shift from 445 to 460 nm, confirming that the fluorescence properties of the N, P-CDs are depend on the surface states of itself rather than the morphology and particle size, and the of the N, P-CDs should be rather uniform.^37,38^Fig. 6C provided the CIE 1931 chromaticity diagram of the N, P-CDs. After calculating from the PL spectrum data under the excitation wavelength of 340 nm, we can get the CIE chromaticity coordinate value of (0.1590, 0.0257), further indicating a blue fluorescence emission property of the synthesized CDs. Fig. 6D shows the PL emission spectra of the N, P-CDs multiply diluted by H_2_O. We can see that the PL emission intensity of the N, P-CDs presents an obvious increase at a lower dilution multiple and then decrease with the continuous increase of the multiple dilution, indicating that the concentration of N, P-CDs is also an important decisive factor for the PL emission performance.

**Fig. 7:**
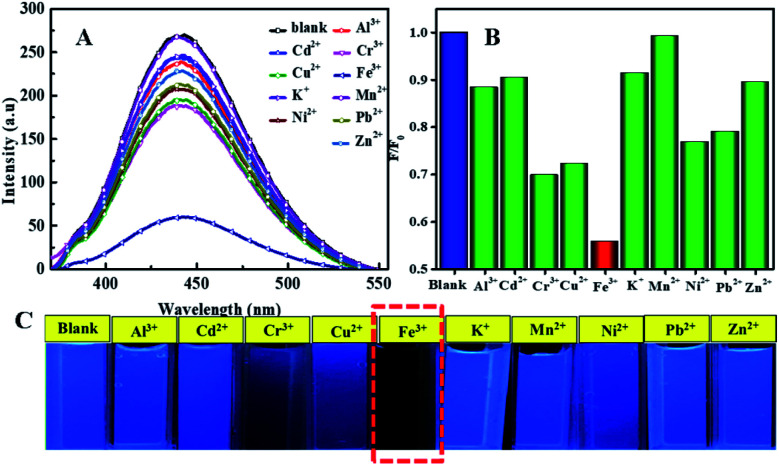
(A) The fluorescence emission spectra of N, P-CDs solution in the presence of various metal ions (B) the corresponding normalized quenched fluorescence emission peak intensity by the above-mentioned metal ions (C) the associated N, P-CDs solution containing metal ions under UV 365 nm. It is noted that the excitation wavelength is 340 nm.

For investigating the selectivity and sensitivity of the as-prepared N, P-CDs to metal ion, the PL spectroscopy of the synthesized N, P-CDs in the presence of various kinds of metal ions with a uniform concentration of 50 μmol L^−1^ was performed. The corresponding PL spectra and relative fluorescence peak intensity (*F*_0_/*F*) are shown in Fig. 7 A and Fig. 7B, respectively. As shown in Fig. 7A and B, all listed metal ions could result in a fluorescence quenching effect to the N, P-CDs. However, Fe^3+^ can lead to a more significant effect to the N, P-CDs on fluorescence quenching. The result demonstrated that the N, P-CDs has a higher selectivity for detection of Fe^3+^ than that of others. Fig. 7C intuitionally illustrates the emission color of N, P-CDs solution containing different metal ions with concentration of 50 μmol L^−1^ under 365 nm excited wavelength. After the addition of 50 μM of Fe^3+^ to the N, P-CDs, the fluorescence color of the N, P-CDs almost disappeared *versus* that of other metal ions, which indicates the high selectivity of as prepared N, P-CDs towards the detection of Fe^3+^ owing to the stronger affinity of Fe^3+^ than the other metal ions. Furthermore, with the aiming at checking the impact of complicated environments on fluorescence quenching of the N, P-CDs as the sensor of Fe^3+^, one category of mixed ions solution including Zn^2+^, Hg^2+^ and K^+^ with concentration of 50 μM for each of the ion were utilized to investigate the fluorescence emission of N, P-CDs with or without the presence of Fe^3+^. As can be seen in Fig. S1,† the coexisting ions, almost similar with the single ions, triggered a slight fluorescence quenching of the N, P-CDs, however, the fluorescence emission of the N, P-CDs were almost completely quenched with an identical level by the Fe^3+^ regardless of the presence of other selected coexisting ions. All of this demonstrated the successful application of fluorescence sensor of the synthesized N, P-CDs for Fe^3+^ detection with a relatively high selectivity.

**Fig. 8:**
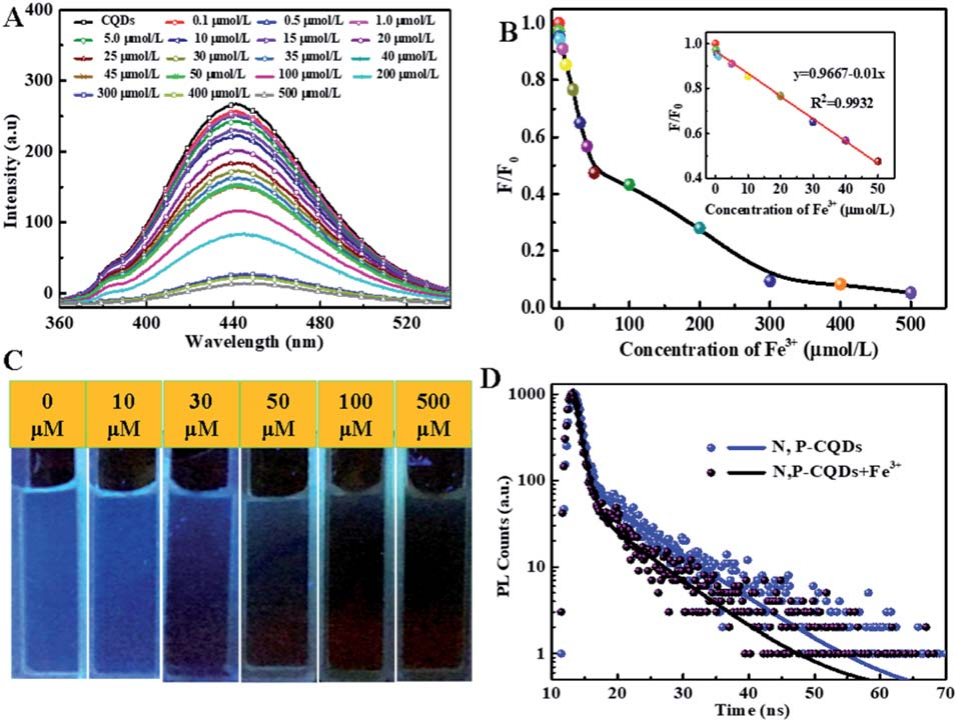
(A) The fluorescence emission spectra of N, P-CDs solution (B) the corresponding normalized quenched fluorescence emission peak intensity (C) the associated N, P-CDs solution (under UV 365 nm) in the presence of varied concentration of Fe^3+^ ions (D) the photoluminescence lifetime decay spectra of N, P-CDs in the absence and presence of Fe^3+^.

To detailedly study the sensitivity of N, P-CDs to Fe^3+^, the fluorescence emission spectra of N, P-CDs with different concentration of Fe^3+^ ranging from 0 to 500 μmol L^−1^ was measured. As exhibited in Fig. 8A, the fluorescence intensity of the N, P-CDs gradually decrease with the increase of Fe^3+^ concentration, indicating that the Fe^3+^ ions can quenched the fluorescence of the N, P-CDs due to interactive chelation between Fe^3+^ and N, P-CDs. Fig. 8B displays the correlation relationship between relative fluorescence intensity (*F*_0_/*F*) of the N, P-CDs and varied Fe^3+^ concentration, where *F*_0_ and *F* are denoted as the fluorescence emission peak intensity of N, P-CDs without and with addition of Fe^3+^, which resulted from the above-mentioned fluorescence emission spectra in Fig. 8A. It well noted that the *F*_0_/*F* decreased with the increase of the Fe^3+^ ions concentration. Furthermore, as exhibited in the inset of Fig. 8B, a good linear relationship can be evidenced between the *F*_0_/*F* of the N, P-CDs and Fe^3+^ concentration in the range of 0–50 μmol L^−1^ with a correlation coefficient (*R*^2^) of 0.9932. And the lowest detection concentration of Fe^3+^ is 0.1 μmol L^−1^, which is not only lower than other reported values for Fe^3+^ detection by bio-mass based CDs, but also much lower than the guideline limit of Fe^3+^ concentration of 5.36 μmol L^−1^ proposed by World Health Organization (WHO).^39–41^ In addition, the fluorescence quenching of the N, P-CDs due to Fe^3+^ addition with various concentration is visible with the naked eye as shown in Fig. 8C. These results suggest that the as-prepared N, P-CDs can sensitively detect Fe^3+^ ions in water even with a very low concentrations (mere 0.1 μmol L^−1^) of. Fig. 8D shows the photoluminescence lifetime decay spectra of the optimal diluted N, P-CDs aqueous in the absence and presence of Fe^3+^. We can observe that the excition lifetime of the N, P-CDs under 340 nm excitation shows a significant reduction from 6.3 ns to 5.1 ns, further confirming that fluorescence of N, P-CDs can be indeed quenched by Fe^3+^ through a dynamic process.^42–44^ At last, we have performed the analytical application of the synthesized N, P-CDs for the detection of Fe^3+^ ion in tap water *via* standard addition recovery method for giving a more comprehensive assessment. It needs to be noted that the tap water was initially treated by 0.22 μm membrane for removing the insoluble particles. The obtained results were summarized in Table 1. Obviously, the recovery rate shows a slight variation from 98.6% to 103.3, verifying the feasibility of Fe^3+^ detection by the N, P-CDs sensor.

**Table 1:** Detection of Fe^3+^ in practical tap water

Water sample	Added Fe^3+^ (μmol L)	detected Fe^3+^ (μmol L)	Error (%)	Recovery (%)
Tap water	0.152	0.157	3.29	103.29
	0.233	0.240	3.00	103.00
	0.857	0.845	−1.40	98.60

**Fig. 9:**
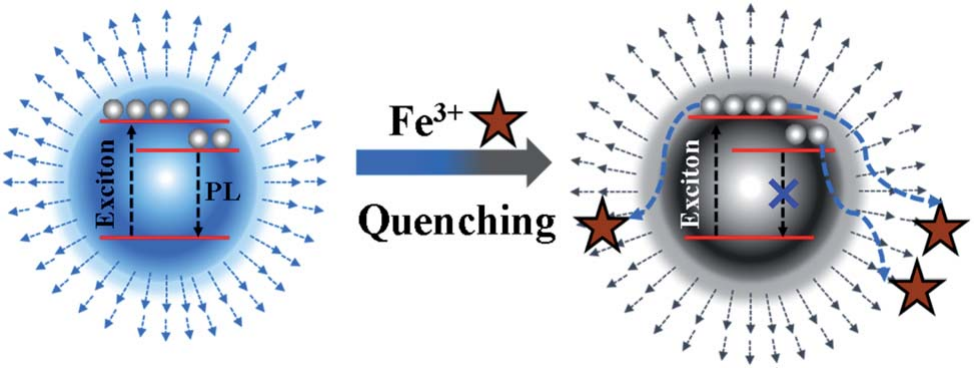
The schematic mechanism of fluorescence emission quenching of the N, P-CDs by Fe^3+^.

Based on the above systematic investigation, a possible mechanism was proposed to elucidate the fluorescence quenching mechanism of N, P-CDs by Fe^3+^ ion, which is shown in Fig. 9. It is well-known that the Fe^3+^ has half-filled 3d orbital which can act as the electron acceptor.^33–35^ On a contrary, the N, P-CDs has sorts of functional groups on the surface of N, P-CDs such as hydroxyl and amino, which can play as the electron donors. These will generate an effective coordination interaction between Fe^3+^ and N, P-CDs, which can make the excited electrons of the N, P-CDs easily transfer into the 3d orbital of Fe^3+^, finally leading to the fluorescence quenching to some extent and meanwhile the exciton lifetime reduction.

## Conclusions

In summary, a kind of N, P-CDs with bright blue fluorescence were successfully synthesized utilizing natural maize starch as the carbon source and phosphate together with urea as the P and N source *via* a facile ethanol solvothermal route. The synthesized N, P-CDs have a morphology of quasi-spherical shape with an average diameter of ∼2.5 nm and exhibit the strongest fluorescence emission peak at 445 nm under the excitation wavelength of 340 nm. Furthermore, such a N, P-CDs can be desirably applied as the fluorescence sensor for selectively and sensitively detecting Fe^3+^ with a detection concentration range from 0.1 to 500 μmol L^−1^. The results indicate that N, P-CDs are an excellent metal fluorescent probe. Therefore, this prepared N, P-CDs are extremely promising for future practical application for the detections of hazardous and toxic ions.

## Conflicts of interest

The authors declare that they have no known competing financial interests or personal relationships that could have appeared to influence the work reported in this paper.

## Supplementary Material

RA-010-D0RA06209J-s001
