# Supplementary material for: Facile synthesis of N, P-doped carbon dots from maize starch via a solvothermal approach for the highly sensitive detection of Fe3+
Source: RSC Adv. 2020 Sep 10;10(55):33483–9. doi: 10.1039/d0ra06209j (PMC9056727; doi:10.1039/d0ra06209j)
Supplement: RA-010-D0RA06209J-s001 [file RA-010-D0RA06209J-s001.pdf]

Electronic Supplementary Material (ESI) for RSC Advances.
This journal is © The Royal Society of Chemistry 2020

Supplementary Information

Facile Synthesis of N, P-doped Carbon Dots from Maize Starch via Solvothermal Approach for the Highly Sensitive Detection of Fe^{3+}

Guohua Dong, [a] Kun Lang, [a] He Ouyang, [c] Wenzhi Zhang, [a,*] Liming Bai, [a] Shijie Chen, [a] Zhuanfang Zhang, [a] Yueyue Gao, [b,*] Zhonghua Mu, [a] and Xiaodan Zhao [a]

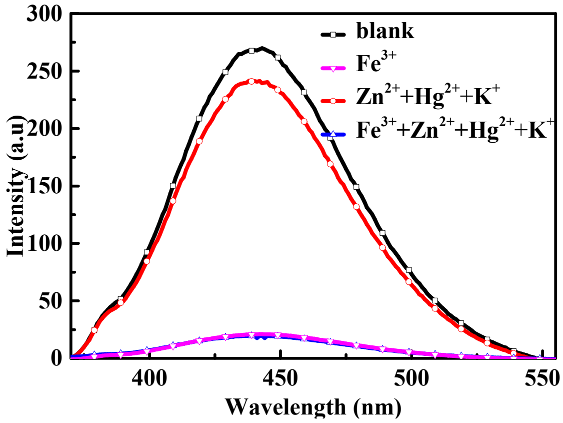

Fig. S1 The fluorescence emission spectra of N, P-CDs solution with or without presence of Fe^{3+} together with other mixed ions solutions.
